# Allergic rhinitis: Disease characteristics and coping measures in Saudi Arabia

**DOI:** 10.1371/journal.pone.0217182

**Published:** 2019-06-26

**Authors:** Abdulmohsin A. Almehizia, Reema K. AlEssa, Khalid M. Alwusaidi, Khalid A. Alzamil, Modhi AlJumah, Sarah Aljohani, Adel F. Almutairi, Mahmoud Salam

**Affiliations:** 1 King Saud Bin Abdulaziz University for Health Science, Riyadh, Saudi Arabia; 2 King Abdullah International Medical Research Center, Riyadh, Saudi Arabia; 3 Imam Saud bin Abdulaziz University, Riyadh, Saudi Arabia; 4 Alfarabi University, Riyadh, Saudi Arabia; National and Kapodistrian University of Athens, SWITZERLAND

## Abstract

**Background:**

Despite allergic rhinitis (AR) being a highly prevalent disease, according to literature, it is often underdiagnosed or undertreated.

**Aim:**

This study explored the disease characteristics of AR in the Saudi community and the non-conventional coping measures used to alleviate symptoms.

**Methods:**

The study was a nationwide cross-sectional study, using a self-report electronic survey distributed via social media networks in 2018. The survey included an explanatory letter and consent. The sample size comprised 3,458 participants and 2,849 adults had at least one of the four signs of AR, i.e. watery-nose, sneezing, nasal obstruction, itchy nose, watery eyes, in the past year, not related to cold/flu. The outcomes of the study were the triggers, pattern, severity and the classification of AR (2016 ARIA guidelines) and coping measures. Descriptive statistics, univariate analytic statistics and binary logistic regression analyses were conducted. The P-value was considered statistically significant at <0.05.

**Results:**

The leading reported trigger of AR was dust (74%, n = 2118), followed by pollen (17%, n = 477), mold (5%, n = 140) and fur (4%, n = 114). The prevalence of intermittent AR was (54%, n = 1,635), while that of persistent AR was (46%, n = 1,314). Almost one-third (34%, n = 959) complained of mild forms of AR, while others complained of moderate to severe forms (66%, n = 1890). The coping measures were mainly shower/humidification 368(12.9%) and herbal hot drinks 266(9.3%). Older participants (adj.OR = 1.3[1.1–1.5]) and overweight participants, (adj.OR = 1.2[1.1–1.4]) reported more persistent forms of rhinitis compared to their counter groups, (adj.P<0.001 and adj.P = 0.032) respectively. Female participants (adj.OR = 0.8[0.7–0.9]) had significantly milder forms of AR, in comparison to males, adj.P = 0.006.

**Conclusion:**

This study presented the disease characteristics of self-reported AR and its associated factors in Saudi Arabia. Special attention should be paid to older age groups and overweight patients who reported persistent forms of AR. Males reported more severe and persistent forms of AR.

## Introduction

Allergic rhinitis (AR) is an inflammatory disease of the nasal mucosa caused by indoor or outdoor allergens [1]. In terms of pathophysiology, immunoglobulin E activates the mast cells or basophils in the nasal mucosa, resulting in the production of vasoactive mediators such as histamine which triggers the inflammation [2]. AR manifests as nasal symptoms (congestion, rhinorrhea, itching, and sneezing) and ocular symptoms (itching, redness and tearing) [3]. Significant complaints also include nasal congestion, and in unilateral presentation, it may suggest the possibility of structural obstruction, such as a polyp, foreign body, or a deviated septum [4]. Since both allergic and non-allergic rhinitis are similar in symptoms, laboratory tests remain the most accurate method in discriminating the type of rhinitis and the nature of allergens. AR may develop in upper airway resistance and impediment of air entry [5] which could progress to comorbidities such as asthma [6].

In persons with AR, there is an abundance of dendritic cells (DCs) that form a network, localized within epithelium and submucosal nasal/upper respiratory tract. In addition, there is an increased number of CD1a1 and CD11c1 DCs in the epithelium and lamina propria of the nasal mucosa clustered with CD41 T lymphocytes and eosinophils. With the presence of an antigen, DCs can polarize naive T cells into either Th1 or Th2 cells according to their own phenotype and with signals received from processed antigens and from the tissue microenvironment during antigen presentation. Within minutes of contact with allergens, the IgE–allergen interaction takes place, leading to mast cell and basophil degranulation and the release of preformed mediators such as histamine and tryptase [7].

The targets of these mediators vary. For example, histamine activates H1 receptors on sensory nerve endings and causes sneezing, pruritus, and reflex secretory responses, but it interacts with H1 and H2 receptors on mucosal blood vessels, leading to vascular engorgement (nasal congestion) and plasma leakage. The symptoms produced immediately after exposure to allergen reach their peak within a few minutes and tend to dissipate within 1 hour. Some individuals continue experiencing symptoms for several hours; others enter a quiescent phase and their symptoms recrudesce after several hours. The nature of the late symptoms is somewhat different from that of the acute symptoms in that sneezing and pruritus are not prominent, whereas nasal congestion is. Overall, these late symptoms occur in approximately 50% of people and, because their relative indolence resembles the clinical presentation of chronic rhinitis, the late phase is of particular scientific interest as a model of chronic allergic disease [7].

Despite the fact that AR is a very prevalent disease, according to literature, it is often underdiagnosed or undertreated by physicians [8]. The prevalence of AR is increasing globally. A report from the World Health Organization (WHO) indicates that 40% of the population suffers from one or more allergic disease [9]. In adults, the prevalence of AR range from 10% to 30%, while in children it is approximately 40% [1, 5]. A study done in the Middle East reported the prevalence of AR symptoms of participants in Western Saudi Arabia as 24%, though only 4% had a prior diagnosis of AR [10]. The prevalence and characteristics of AR among Saudi adults remains undetermined.

The burden of AR on patients is significant; it negatively affects their social life, school performance, work productivity, and the quality of sleep [6]. Treatment options of AR include avoidance of exposure to allergens, pharmacological treatment of symptoms such as antihistamines and/or corticosteroids, and allergen immunotherapy [11]. However, AR patients often resort to unconventional or non-pharmacological treatments to alleviate their symptoms or facilitate coping with the AR exacerbation. Though these interventions are extensively disseminated by alternative medicine experts, social media and/or the community, the effectiveness of these coping measures remains subjective, since they are based on self-experimentation and perceived benefits.

Despite the spread of AR worldwide and in particular within high risk countries such as Saudi Arabia, there is a dearth of studies investigating disease characteristics and local coping measures [12]. Saudi Arabia is known for its frequent and periodic sandstorms in all seasons. Sandstorms transport many types of microorganisms and dust particles that can trigger or exacerbate respiratory diseases such as AR and asthma [13]. This study aimed at determining the prevalence of disease characteristics among patients complaining of AR, as well as exploring the coping measures adopted during an exacerbation. These findings will provide insight regarding AR for the Saudi population, clinicians and researchers. It will also provide guidance to expatriate employees planning to reallocate to Saudi Arabia, a high income country and a magnet for job opportunities.

## Materials and methods

### Study design

The study had a cross-sectional design.

### Study setting

Between July 15 and August 7, 2018, an anonymous, Arabic language electronic survey was distributed nationwide through social media networks. These networks were mainly those attracting general public interest, for example community health, sports, political shows, entertainment to a smaller degree social groups or gatherings. A rapid growth in the usage of internet has been witnessed in Saudi Arabia between 2000 and 2012, where more than 84% are internet users [14, 15]. In 2018, the percentage of Saudi Arabian residents accessing the social media was 91% as per the Global Median Insight among whom users of Twitter are estimated to be 17 million users (52%) and those using WhatsApp were 24 million users (73%) of the total population [16].

This convenient sampling method maximized the exposure of the survey to participants from the various regions in Saudi Arabia and boosted the representativeness of the sample. The distribution of the survey was done in three rounds. Incomplete questionnaires were dropped out. It included an explanatory letter that explained the purpose of the study and clarified some of the clinical terms such as the AR sign and symptoms. Participants were requested to report their nationality and region upon submission.

### Study subjects

Eligibility criteria included adults who had at least one of the four signs of AR, i.e. a watery runny nose, sneezing (violent/in bouts), nasal obstruction (inability to breath), itchy nose; watery/red itchy eyes, during the past year and not related to an episode of cold/flu [17]. Participants were asked if their symptoms were exacerbated directly after an exposure to one of the four triggers (dust; pollen; fur; mold). A discrepancy between the prevalence of the self-reported and the clinically confirmed AR has been reported by Huang et al. 2018 [18]. In Saudi Arabia, the prevalence of self-reported AR has not been previously reported. Accordingly, the sample size was calculated as 2,400, based reported prevalence of self-reported AR of 46.77% in one Asian country [18], a margin of error of 2% and a 95% confidence level. Taking into account the method of data collection and risk of incomplete surveys, the number of invited participants was increased by 25%, and multiple distributions were launched throughout social media networks. Participation was not restricted to Saudi citizens and the job market in Saudi Arabia accommodates expatriate workers from all over the world, so the data collected is not only representative of the country itself. Any participant who reside outside the targeted setting was excluded.

### Data collection

The data collection tool comprised of two main domains. It was developed based on the commonly reported exposures in literature (participants’ characteristics) [12, 19, 20] and the 2016 ARIA guidelines that defined the AR disease characteristics [21]. The participants’ characteristics were the study exposures, which included the sex, age (years), body mass index (BMI), area of residence, occupation, the presence of chronic medical conditions, disease duration, family history, and smoking status. Age was categorized into younger adults 18–24 years (millennial generation) most of whom grew up with access to digital communication and older adults (≥25 years) [22]. The study outcomes were three disease characteristics (triggers, pattern, severity). Reported triggers allergens were accounted as mutually exclusive i.e. pollen, mold, furred animals, or dust. Participants who reported more than one type of trigger were dropped out. As per ARIA guidelines, the triggers of AR are either pollen, house dust mites or both [21]. However, authors in this study referred to the general term “dust” which includes house mites, indoor and outside dust particles, as the public community might not be able to differentiate dust from house mites that are too small in size to be recognized by the naked eye [23]. ARIA recently classified the pattern of AR based on the reported duration of symptoms; as intermittent or persistent. Intermittent AR lasts for ≤4 days per week for less than four weeks, whereas persistent AR lasts for >4 days per week for more than four weeks [21, 24]. Patients with intermittent AR experienced sneezing, eye symptoms, and watery secretions; while patients with persistent AR have seromucous secretions, postnasal drip, smell disturbances, nasal obstruction and may be associated with asthma and chronic sinusitis [25]. The severity (mild vs. moderate-severe) of AR is based on the symptoms and its impact on the quality of life. Mild symptoms neither interfere with sleeping nor cause impairment of daily activities, such as sports, leisure, or work performance. Moderate to severe symptoms affect at least one of the previously stated life aspects [26]. Unconventional coping methods during AR attacks included a variety of reported measures adopted by the participants to alleviate their symptoms, which they perceived as beneficial.

The English and Arabic versions of the survey are presented in English version (S1 Survey) and Arabic version (S2 Survey). The data collection tool was sourced from the previously validated ARIA guidelines. Back-to-back language translation (English-Arabic) was conducted by two individuals to insure the operational definitions retained their intended meaning. Then the data collection tool was pilot tested on a group of nine persons who recommended minor modifications on some terms such as collapsing the answers of the marital status question (separated vs. widowed), and replacing the actual income variable with financially comfortable vs. uncomfortable. According to the subjective comments collected during the pilot phase, some of the Arabic terms such as mold, dust, pollen, runny nose, etc. were also modified so that they can be easily comprehended by the local targeted community.

### Data management and analysis

Data were entered and analyzed using SPSS statistical software (version 25, IBM, NY, USA). Descriptive statistics such as the mean, standard deviation, frequency, and percentages were used to present the participants’ and disease characteristics. Disease characteristics were analyzed as categorical variables using Pearson’s Chi-square. Two binary logistic regression analyses were conducted to determine factors associated with study outcomes and control for any potential confounding effect. The adjusted Odds Ratio and 95% confidence interval (adj.OR[95%CI)] were presented and tabulated, with a P-value statistically significant at <0.05. The sample was later stratified by the smoking status, to determine if smoking had a confounding effect on the pattern and severity of self-reported AR (S1 Dataset).

### Ethical consideration

A self-explanatory letter of invitation to participate was presented electronically to each of the participants. All participants had given informed consents for their participation. Participants consented by ticking “agree”, indicating their agreement to provide their feedback for this research study. The Institutional Review Board of the Saudi Ministry of National Guard Health Affairs, Riyadh, Saudi Arabia (Protocol # RSS18-019R), approved this study. Studies conducted followed the recommendations of the International Conference on Harmonization for Good Clinical Practice and the Uniform Requirements for manuscripts submitted to biomedical journals.

## Results

### Participant, disease characteristics and coping measures

Participants with self-reported symptomology of AR were (82.4%, n = 2,849). The majority of participants 2,786(97.8%) had at least two signs of AR (including ocular symptoms), while 63(2.2%) reported only watery/red itchy eyes which might indicate a co-exiting allergic conjunctivitis. The highest prevalence was in Central Saudi Arabia (53.4%), followed by the Western region (22.1%), the Eastern region (12.4%) and both the Northern/Southern regions (12.1%). Of the 2,849 cases, 59.4% were females with 40.6% males, with a mean±standard deviation of age 29±10 years. Almost 6% were underweight (BMI<18.5), 38% had a normal weight (BMI = 18.5–24.9) and 58% pre-obese/obese (BMI≥25). Smokers comprised 13.2% of the sample, and almost 37% had at least one chronic medical condition. Almost 55% admitted to complain of snoring, followed by allergy/asthma (37.2%) and irritable bowel syndrome (23.7%). The age of onset of symptoms was dropped out due to extensive recall bias and lack of accuracy (answers were mainly since childhood or don’t recall). Two thirds of participants (64.1%) had a positive family history of allergic rhinitis (Table 1).

**Table 1:** Prevalence of allergic rhinitis.

	n(%)
	2849(100%)
**Sex**	
Male	1158(40.6%)
Female	1691(59.4%)
**Age(years)**	
<25	1351(47.4%)
≥25	1498(52.6%)
Mean±Standard deviation	29.22±10.1
**BMI**	
Underweight(<18.5)	160(5.6%)
Normal(18.5–24.9)	1073(37.7%)
Preobese(25–29.9)	795(27.9%)
Obese(≥30)	821(28.8%)
Mean±Standard deviation	26.34±5.9
**Smoker**	
Yes	377(13.2%)
No	2472(86.8%)
**Chronic Medical condition**	
None	1788(62.8%)
Yes	1061(37.2%)
Snoring	584(55.0%)
Allergy/Asthma	399(37.6%)
Irritable bowel syndrome	251(23.7%)
Septum deviation/Sinusitis	215(20.3%)
Hypertension/Cardiac	97(9.1%)
Diabetes	86(8.1%)
Hypothyroidism	53(5.0%)
Anemia	52(4.9%)
Dyslipidemia	35(3.3%)
VitD deficiency	19(1.8%)
Hepatitis B	6(0.6%)
Gout	1(0.1%)
**Family history of Allergic Rhinitis**	
Yes	1827(64.1%)
No	375(35.9%)

n: frequency, %: percentage

**Fig 1:**
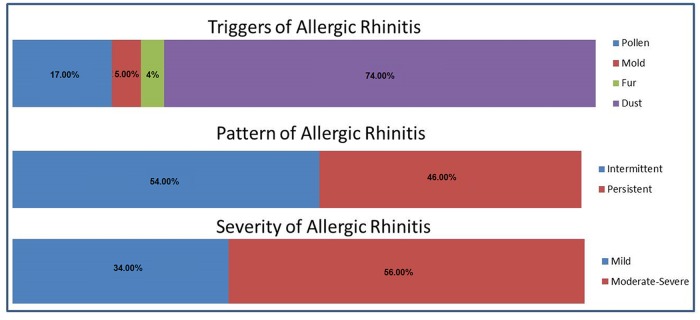
Allergic rhinitis characteristics.

Triggers of AR, pattern, and levels of severity are presented in Fig 1. The leading trigger of AR was dust (74%), followed by pollen (17%), mold (5%) and fur (4%). The pattern of AR was equally distributed and more than half of the sample (56%) complained of moderate to severe AR.

In terms of conventional therapies, more than half of the participants took antihistamine pills (55.1%), followed by a visit to health care clinics (1.9%) and usage of inhalers (1.1%). The most frequently reported unconventional coping measures were shower/humidification (12.9%) and herbal hot drinks (9.3%). Other types of measures are enlisted in a descending order (Table 2).

**Table 2:** Conventional and unconventional AR coping measures.

	n(%)*
**Conventional therapies**	
Antihistamines pills	1569(55.1%)
Visit clinics	55(1.9%)
Inhalers	31(1.1%)
Vitamin C	16(0.6%)
Steroids	9(0.3%)
**Unconventional therapies**	
Shower/humidification	368(12.9%)
Herbal hot drinks	266(9.3%)
Citrus juices/vinegar	99(3.5%)
Honey bee wax	47(1.6%)
Lotions/oil rubs/vapors	38(1.3%)
Sleeping	33(1.2%)
Saline sprays	28(1.0%)
Antibiotics	19(0.7%)

n: frequency, %: percentage;

*:non-mutually exclusive

### Factors associated with AR triggers

Males were more likely (20.6%) to have pollen as a trigger for AR, in comparison to females (14.1%, P<0.001). On the contrary, females were more likely (77.8%) to complain of AR triggered by dust, compared to males (69.3%, P<0.001). Older age group were also more likely to have AR triggered by pollen (19.8%), compared to younger participants (13.4%, P<0.001). Younger AR participants were more likely to be affected by dust (77.7%), compared to their counter group (71.3%, P<0.001). BMI and a family history of AR showed no statistically significant differences across the type of triggers. However, being a smoker was associated with AR triggers such as pollen (20.7%, P = 0.028) and furred animals (6.1%, P = 0.026), while non-smokers were more affected (75.3%, P = 0.003) by dust. Persistent AR was associated with mold (7.2%, P = 0.01). Moderate to severe AR was associated with pollen triggers (18.0%, P = 0.012), with milder forms of AR being associated with dust as a trigger (77.6%, P = 0.005) (Table 3).

**Table 3:** Association between trigger of allergic rhinitis and sample characteristics.

	Pollen	Mold	Furred animals	Dust
	n(%)	n(%)	n(%)	n(%)
**Sex**				
Male	239(20.6%)	66(5.7%)	50(4.3%)	803(69.3%)
Female	238(14.1%)	74(4.4%)	64(3.8%)	1315(77.8%)
	χ2 = 21.2,P<0.001*	χ2 = 2.8,P = 0.108	χ2 = 0.5,P = 0.476	χ2 = 25.6,P<0.001*
	RR[95%] = 0.6[0.5–0.8]	RR [95%] = 0.8[0.5–1.1]	RR[95%] = 0.9[0.6–1.2]	RR[95%] = 1.5[1.3–1.8]
**Age(years)**				
<25	181(13.4%)	57(4.2%)	63(4.7%)	1050(77.7%)
≥25	296(19.8%)	83(5.5%)	51(3.4%)	1068(71.3%)
	χ2 = 20.6,P<0.001*	χ2 = 2.6,P = 0.103	χ2 = 2.9,P = 0.087	χ2 = 15.3,P<0.001*
	RR[95%] = 1.56[1.3–1.9]	RR[95%] = 0.6[0.5–1.0]	RR[95%] = 0.7[0.5–1.1]	RR[95%] = 0.7[0.6–0.8]
**BMI**				
Underweight/Normal	196(15.9%)	55(4.5%)	49(4.0%)	933(75.7%)
Preobese/Obese	281(17.4%)	85(5.3%)	65(4.0%)	1185(73.3%)
	χ2 = 1.118,P = 0.290	χ2 = 0.9,P = 0.328	χ2 = 0.004,P = 0.948	χ2 = 2.0,P = 0.157
	RR[95%] = 1.14[0.9–1.4]	RR[95%] = 1.2[0.8–1.7]	RR[95%] = 1.0[0.7–1.5]	RR[95%] = 0.9[0.7–1.0]
**Positive Family history**				
Yes	321(17.6%)	90(4.9%)	74(4.1%)	1342(73.5%)
No	57(15.2%)	18(4.8%)	19(5.1%)	281(74.9%)
	χ2 = 1.229,P = 0.134	χ2 = 0.010,P = 0.459	χ2 = 0.010,P = 0.458	χ2 = 0.010,P = 0.458
	RR[95%] = 1.2[0.9–1.6]	RR[95%] = 1[0.5–1.8]	RR[95%] = 1[0.5–1.8]	RR[95%] = 1[0.5–1.8]
**Smoker**				
Yes	78(20.7%)	19(5.0%)	23(6.1%)	257(68.2%)
No	399(16.1%)	121(4.9%)	91(3.7%)	1861(75.3%)
	χ2 = 4.9,P = 0.028*	χ2 = 0.2,P = 0.903	χ2 = 4.9,P = 0.026*	χ2 = 8.7,P = 0.003*
	RR[95%] = 1.4[1.0–1.8]	RR[95%] = 1.0[0.6–1.7]	RR[95%] = 1.7[1.0–2.7]	RR[95%] = 0.7[0.6–0.9]
**Pattern of rhinitis**				
Intermittent	254(16.5%)	45(2.9%)	65(4.2%)	1171(76.3%)
Persistent	223(17.0%)	95(7.2)	49(3.7%)	947(72.1%)
	χ2 = 0.091,P = 0.763	χ2 = 28,P = 0.010*	χ2 = 20.5,P = 0.493	χ2 = 6.6,P = 0.100
	RR[95%] = 1.0[0.8–1.3]	RR[95%] = 2.9[1.8–3.7]	RR[95%] = 0.9[0.6–1.3]	RR[95%] = 0.8[0.7–0.9]
**Severity of rhinitis**				
Mild	137(14.3%)	41(4.3%)	37(3.9%)	744(77.6%)
Moderate to severe	340(18.0%)	99(5.2%)	77(4.1%)	1374(72.7%)
	χ2 = 6.261,P = 0.012*	χ2 = 1.3,P = 0.261	χ2 = 0.7,P = 0.781	χ2 = 7.91,P = 0.005*
	RR[95%] = 1.3[1.1–1.6]	RR[95%] = 1.2[0.9–1.7]	RR[95%] = 1.0[0.7–1.6]	RR[95%] = 0.8[0.6–0.9]

n: frequency, %: percentage, χ2: Pearson Chi-square test, df: degree of freedom, RR: relative risk, [95%]: 95% confidence interval,

P: P-value

*: P-value statistically significant at <0.05.

### Factors associated with the pattern and severity of rhinitis

Persistent forms of AR were more prevalent in the older age group (49.8%) and pre-obese/obese participants (48.6%), (P<0.001 and P = 0.003 respectively). Moderate-severe forms of rhinitis was more prevalent among male participants (70.7%), older age group (68.6%), and smokers (74.0%), (P<0.001, P = 0.008, and P = 0.001 respectively) (Table 4).

**Table 4:** Association between pattern and severity of allergic rhinitis with sample characteristics.

	Pattern of rhinitis		Severity of rhinitis	
	Intermittent	Persistent	Mild	Moderate/Severe
	n(%)	n(%)	n(%)	n(%)
**Sex**				
Male	628(54.2%)	530(45.8%)	339(29.3%)	819(70.7%)
Female	907(53.6%)	784(46.4%)	620(36.7%)	1071(63.3%)
	χ2 = 0.098,P = 0.755		χ2 = 16.810,P<0.001*	
	RR[95%] = 1[0.9–1.2]		RR[95%] = 0.7[0.6–0.8]	
**Age (years)**				
<25	783(58.0%)	568(42.0%)	488(36.1%)	863(63.9%)
≥25	752(50.2%)	746(49.8%)	471(31.4%)	1027(68.6%)
	χ2 = 17.200,P<0.001*		χ2 = 6.966,P = 0.008*	
	RR[95%] = 1.4[1.2–1.6]		RR[95%] = 1.2[1.1–1.4]	
**BMI**				
Underweight/Normal	704(57.1%)	529(42.9%)	436(35.4%)	797(64.6%)
Preobese/Obese	831(51.4%)	785(48.6%)	523(32.4%)	1093(67.6%)
	χ2 = 9.058,P = 0.003*		χ2 = 2.813,P = 0.093	
	RR[95%] = 1.3[1.1–1.5]		RR[95%] = 1.1[1.0–1.4]	
**Family history**				
Yes	973(53.3%)	854(46.7%)	586(32.1%)	1241(67.9%)
No	185(49.3%)	190(50.7%)	130(34.7%)	245(65.3%)
	χ2 = 1.921,P = 0.080		χ2 = 0.952,P = 0.164	
	RR[95%] = 1.2[0.9–1.5]		RR[95%] = 0.9[0.7–1.1]	
**Smoker**				
Yes	202(53.6%)	175(46.4%)	98(26.0%)	279(74.0%)
No	1333(53.9%)	1139(46.1%)	861(34.8%)	1611(65.2%)
	χ2 = 0.15,P = 0.901		χ2 = 11.435,P = 0.001*	
	RR[95%] = 1[0.8–1.3]		RR[95%] = 1.5[1.2–1.9]	

n: frequency, %: percentage, χ2: Pearson Chi-square test, df: degree of freedom, RR: relative risk,[95%]: 95% confidence interval, P: P-value

*: P-value statistically significant at <0.05.

Binary logistic regression analyses showed that older AR participants (≥25) and overweight AR participants were significantly 1.3[1.1–1.6] and 1.2[1.1–1.4] times more likely to complain of persistent rhinitis compared to their counter age group, (adj.P<0.001 and adj.P = 0.032) respectively. Female AR participants 0.8[0.7–0.9] were significantly less likely to be associated with moderate-to-severe forms of AR, adj.P = 0.006 (Table 5).

**Table 5:** Factors significantly associated with pattern and severity of allergic rhinitis.

	Pattern of rhinitis			Severity of rhinitis		
	Interment 0; Persistent 1			Non-Mild 0; Moderate-Severe 1		
	B(S.E.)	Adj.OR,[95%CI]	Adj.P value	B(S.E.)	Adj.OR,[95%CI]	Adj.P value
**Sex**						
Female vs. Male	0.10(0.08)	1.1[0.9–1.3]	0.219	-0.24(0.09)	0.8[0.7–0.9]	0.006*
**Age (years)**						
≥25 vs. <25	0.29(0.08)	1.3[1.1–1.6]	<0.001*	0.14(0.08)	1.1[1.0–1.3]	0.105
**BMI**						
Pre-obese/Obese vs. Underweight/Normal	0.17(0.08)	1.2 [1.1–1.4]	0.031*	0.04(0.08)	1.0[0.9–1.2]	0.641
**Smoker**						
Yes vs. No	0.02(0.12)	1.0[0.8–1.3]	0.898	-0.26(0.13)	0.8[0.6–1.0]	0.053

B: beta, SE: standard error, OR: odds ratio, adj: adjusted, [95%CI]: 95% confidence interval, P: P-value

*: P-value statistically significant at <0.05

Furthermore, the sample was stratified based on being a smoker or not, to determine if smoking had a certain confounding role on the pattern or severity of AR. Among the non-smoker group, older participants 1.4[1.2–1.6] and heavier participants 1.2[1.1–1.4] reported more persistent forms of AR, adj.P<0.001 and adj.P = 0.04 respectively. Female participants who were non-smokers 0.8[0.7–0.9] reported milder forms of AR, adj.P = 0.009. However, among the smoker participants who reported AR, no statistically significant variables were observed.

## Discussion

This epidemiological study presents to the public and healthcare community the the patient and disease characteristics of 2,849 participants complaining of AR in a Middle Eastern country with a relatively harsh climate. While the Prick test remains the most accurate diagnostic test to confirm an allergic trigger, there was a need to launch a less costly preliminary community surveillance to evaluate the general status of AR. Prick tests range in cost between $60-$300 and for research purposes; participants need to be followed-up for further observation. However, at this stage results can be generalized to settings with similar environmental conditions. In addition, it provides insight regarding AR for expatriates who are seeking job opportunities and willing to relocate to Saudi Arabia, as well as those visiting this country on a temporary basis. A multisite study conducted in five Middle Eastern countries revealed that only 10% of the 7,411 self-reported AR cases were medically diagnosed with AR [27]. Based on these findings, future diagnostic studies may follow to confirm the true clinical association between AR triggers and severity of symptoms.

The leading self-reported triggers of AR in this setting was dust, followed by pollen, which were different from triggers reported in other Asian countries (dust and animal dander) [28]. In Southwestern Iran, the leading outdoor allergen was weeds (89%), tree and grass, while indoor allergens were mainly mites (43%) [29]. Authors believe that it is possible within the same region, the prevalence and severity of AR may vary. A study conducted in Southern Saudi Arabia reported that 43% of their participants had severe AR [30] which is less than the current study. A review paper highlighted that severe forms of AR ranged between 1% and 65.9% [31]. For example, people residing close to construction areas, green parks or farms, or those raising pets indoors may exhibit variations in the same geographical region. Sever AR symptoms was reported to limit work/school activities in 72% and cause absenteeism in 35% of AR patients within a 12 months period [27]. AR is apparently more common in the whole Middle East region as elsewhere in the world, and AR patients suffer from their symptoms on all or most days during the times of the year [27]. The prevalence and severity of AR do not only vary between regions, but is also escalating. A regional study reported that the prevalence of AR increased from 20% to 25% during a nine year period [32] which can be attributed to global warming and sources of pollution. Investigating the pattern and severity of AR across regions may not be contributing to the science body of knowledge, in comparison to investigating the proximal environmental characteristics.

The whole region is a common route for sand storms, a natural hallmark of the country [20]. During the hot season (May to October) residents, remain indoors in air-conditioned facilities (houses, offices and shopping centers). Residing in dry climates such as the Saudi inland regions could be an environmental factor that contributes to nasal mucosal dryness. Previous studies provided evidence that sandstorms were the main factor causing allergic and non-allergic AR in this country [13], as well as other Asian settings [33]. However, the authors propose that the sandstorms are not the trigger itself, as conditioned airs, in-door furniture, house mites, carpets and/or vegetation all accumulate dust. AR associated with dust was more reported by females and younger age group. It is worth mentioning that house dust mites might not be distinguished from the indoor/outdoor dust particles by the public community. House dust mites are microscopic eight-legged creatures that exist in dust, mostly found in bed sheets, carpets, furniture and cloth toys [23]. For instance, a 1gram of dust may contain 10,000 mites that mainly feed on the outer layer of human and animal skin. Detecting house mite allergens require environmental screening and Prick tests. Therefore, the public community shouldn’t overlook any source of indoor dust, as there is a high chance it might be infested with mites.

Authors believe that some participants who complained of AR spend more time outdoors making them at higher risk of exposure to pollen released from certain types of dessert plants. AR triggered by pollen was reported by participants to be moderate to severe, in comparison to that triggered by dust which was reported to be mild. The botanical fauna in Saudi Arabia is pretty distinctive compared to other habitats, being able to tolerate the harsh dessert conditions. The severity of AR associated with pollen triggers was comparable to a study stating that 18.9% of patients exposed to pollen grains developed severe AR [34]. In other words, using a skin prick test and starting at baseline, a rise of 60 grass pollen grains/m3 increases the risk of suffering from a severe AR by 8% after adjusting for potential confounders [35]. A second study confirmed this findings that more severe forms of AR were prevalent among those exposed to pollen compared to house dust [36]. Surprisingly, one Japanese study confirmed a relationship between the level of airborne pollen during certain seasons and suicidal mortality among females [37]. In this setting, males were significantly more prone to suffer from moderate to severe forms of AR, possibly due to, in comparison to females, being affected by pollen as a trigger of AR, known to cause severe AR [31]. It is worth mentioning that the increased use of ornamental plants in parks and gardens, public and work places and houses may have created new sources of pollen aeroallergens [38]. The use of face masks, readily available and accessible, is recommended as essential protection from pollen during high seasons.

AR triggered by mold tends to be persistent, as mold exists mainly indoors and in places unnoticed by residents. Occupants of damp and/or moldy buildings most frequently and persistently complain of cough, rhinosinusitis, worsening of asthma, and increased susceptibility to respiratory infections [39]. The change in the living life style from an open air type of environment (e.g. farms) to energy efficient, tight building type of environment, made people spend up to 90% of their time indoors [40]. The CDC stated that mold can grow on any damp or moist saturated surface, and suggested that early exposure to it is an indicative of asthma development [41]. Due to hot weather, continuous closure of windows and air conditioning in this setting, houses may generally lack the much needed ventilation which makes the presence and exposure to mold persistent. However, some studies that have tried to relate AR symptoms with spore levels from air samples have found little correlation with the incidence or severity. However, in experimental mice studies, some types of mold spores (satratoxin) caused severe intraalveolar, bronchiolar, and interstitial inflammations. The severity of inflammation was dose related and dependent on whether the spore strains were either toxic or not. Similar findings were observed between the high doses of the nontoxic molds strains and the lower dose of the toxic strain. The lower dose of the nontoxic strains did not induce observable inflammation [39].

The least reported AR trigger was furred animals (4%). The popularity of cats and dogs as pets affects the rates of AR among the general population. For instance, in Europe the frequency of cat ownership ranges from 7.2 to 35% and dog ownership from 5.4 to 35%, while in the United States, nearly 40% and 33% of households own dogs and cats, respectively. The overall European cat sensitization rate was 26.3% and for dog allergens, it was 27.2%, ranging from 16.1 to 56% [42]. Although the Saudi Arabian community supports its nomadic traditions of raising cattle in outdoor ranches, the authors believe that their exposure is lower than indoor pets. Unlike other settings where domestic dogs and cats are raised abundantly, the Saud Arabian population is religiously oriented and the possession of dogs is not favored. This may justify why the minority of the targeted population reported AR to furred animals. Authors noticed that furred animals were mainly associated with more severe forms of AR. In contrast, smoking was a variable associated with AR triggered by both pollen and furred animals, but not with dust. Smoking irritates airways, impairs its mucosal lining and it has been associated with AR, especially among passive smokers [43]. Although one study reported that current smokers had a lower risk of AR [44], smoking was not associated with the reported pattern or severity AR in this setting.

A study reported that the prevalence of intermittent AR in Saudi Arabia was 65%, in comparison to the persistent form of AR (35%) [45]. In this study, the prevalence of persistent AR as reported by study participants was lower, and was associated with older age and overweight groups. The age of almost 95% of the study participants in this study ranged between 18 and 49 years who are usually the predominant users of social media networks. While some might argue that this might be an over-representation of younger adults, it is a known fact that the prevalence of AR rises through childhood and adolescence to affect substantial proportion of the individuals in young adulthood [46]. Moreover, it was reported that a positive association existed between obesity and AR in younger adults, yet among adults being overweight was not associated with AR [19]. Previous studies also stated that older adults often experience dryness in the mucus membranes and/or nasal congestion which often exacerbates into persistent forms of rhinitis [47]. Persistent forms of AR have been reported to be associated with 43.1% of moderate-to-severe rhinitis, while intermittent forms were associated with 56.9% of intermediate severity AR conditions [48]. Authors believe that the severe forms of AR are probably due to the participant’s unexpected encounter with certain AR triggers. In other words, though people with AR are usually extra vigilant and take precautions with AR triggers, their exposure was probably accidental or beyond control. Their symptoms might have been triggered and deteriorated due to the absence of their usual safety measures or treatment.

One of the main outcomes of the study was to determine the coping measures participants adapted to relief AR symptoms. Responses were diverse, yet upon categorizing, the most frequently reported measure related to humidification such as taking a shower. A review paper of the so called “neglected non-pharmacological treatments” of AR recommended by two Persian pioneers Rhazes and Avicenna showed that avoiding overeating and polydipsia, massage of the lower extremities, adjusting the duration and time of sleep, sleeping in the supine position, avoiding exposure of the head to cold air and taking a shower early in the morning all aided in managing AR exacerbations [49]. Nasal spray use is common among AR patients though they do not provide the same warm soothing and moist humidification experienced in bathing. During bathing allergens in contact with the body surface and nasal cavity are washed away, removing the AR trigger that relieves the symptoms. A study recommended indoor humidifiers to remove indoor allergens such as dust mites [50]. Herbal drinks were the second option as a coping measure including a variety of herbs, such as green tea, mint, and thyme. To date, there is scarcity of evidence related to the effectiveness of such non-conventional methods. The authors propose that since each of the self-reported measures has been proven effective from the participants’ point of view, future interventional studies are worth conducting to test their efficacy.

### Limitations

AR is a manifestation of an immunological reaction, and its etiology is probably linked to both genetic predispositions and environmental influences. The scope of this study was beyond a genetic investigation. However, many of the gene-based AR studies revealed no significant association with AR [51]. Allergic and non-allergic rhinitis are both similar in signs and symptoms, except that non-allergic rhinitis don’t involve the immune system. Though authors reminded the participants to report their symptoms that exacerbated after an exposure to one of the four triggers, Prick allergy and blood tests are only capable of discriminating between the two forms of rhinitis. The retrospective nature of the study design may have been prone to a certain degree of recall bias, especially when participants were asked about the disease characteristics and coping measures. However, authors propose that AR patients are expected to be fully aware of such information, due to the chronic nature of AR. In addition, web-based surveys may be subject to a misunderstanding of some operational definitions. AIRA guidelines specified an additional type of AR which is the occupational AR[21], yet it was not assessed in this study. Moreover, authors failed to obtain accurate timing of the date of onset of the disease due to recall biases. Environmental characteristics, such as nearby vegetation, type of air conditions, presences/absence of carpets, etc. were not assessed, as authors fear it may add length to the survey and risk incomplete submissions of questionnaires. Finally yet importantly, there is no doubt that the usage of social networks might be less common among the older generations, though AR emerges at younger ages.

## Conclusions

This study presented the disease characteristics and associated factors of AR as reported by the public community in Saudi Arabia. AR was mainly triggered by dust, followed by pollen, mold and fur. More than half of the AR cases in Saudi Arabia suffered from moderate to severe symptoms and intermittent pattern of the disease. Factors associated with triggering AR by pollen were male gender, older age group, smoking and persistent AR. AR triggered by mold was associated with persistent forms of the disease, while AR triggered by fur was associated with smoking. AR triggered by dust was associated with the female gender, younger age, smoking, as well as, mild forms of the disease. Patients with AR are advised to adopt weight control measures as they were more likely to endure persistent forms of the disease if overweight.

## Supporting information

S1 Survey English language version of the survey. (DOCX)

S2 Survey Arabic language version of the survey. (DOCX)

S1 Dataset Dataset. (SAV)
