# Supplementary material for: Allergic rhinitis: Disease characteristics and coping measures in Saudi Arabia
Source: PLoS One. 2019 Jun 26;14(6):e0217182. doi: 10.1371/journal.pone.0217182 (PMC6594581; doi:10.1371/journal.pone.0217182)
Supplement: S1 Survey — (DOCX) [file pone.0217182.s001.docx]

**Triggers, pattern and severity and coping mechanisms of allergic rhinitis in Saudi Arabia**

1. Age (years): __________
2. Sex:

- Male
- Female

1. Weight:
2. Height:
3. Nationality:

- Saudi
- Non-Saudi (Specify:___________)

1. Area of residence: __________
2. Marital Status:

- Married
- Single
- Separated/Widowed

1. Educational level:

- Non-educated
- School level
- University level

1. Monthly income:

- Financially comfortable
- Financially uncomfortable

1. Do you suffer from any medical conditions? _____________________________
2. Do you complain of snoring?

- No
- Yes
- Don’t know

1. Are you a smoker?

- No
- Yes, how many cigarettes per day ( __ )

| **All questions are about problems which occur when you DO NOT have a cold or the flu.**  **In the past year:** | | | | | | | | | | | | | | | | | | |
| --- | --- | --- | --- | --- | --- | --- | --- | --- | --- | --- | --- | --- | --- | --- | --- | --- | --- | --- |
| 10. | **Do you have any of the following symptoms or have a history of suffering from any of them** | | | | | | | | | | | | | | | | | |
|  | 1. Watery runny nose | | | | | | | | | | | | - Yes | | | | - No | |
|  | 1. Sneezing (especially violent and in bouts) | | | | | | | | | | | | - Yes | | | | - No | |
|  | 1. Nasal obstruction (feeling of being unable to breathe through your nose) | | | | | | | | | | | | - Yes | | | | - No | |
|  | 1. Itchy nose | | | | | | | | | | | | - Yes | | | | - No | |
|  | 1. Watery, red itchy eyes | | | | | | | | | | | | - Yes | | | | - No | |
| 11. | What causes your symptoms? | | | | | | | | | | | | | | | | | |
|  | 1. Pollen from trees, flowers and grass | | | | | | | | | | | |  | | | | | |
|  | 1. Mold (both indoors and outdoors) | | | | | | | | | | | |  | | | | | |
|  | 1. Furred animals (esp. cats, dogs and mice) | | | | | | | | | | | |  | | | | | |
|  | 1. Dusty places | | | | | | | | | | | |  | | | | | |
| 12. | How long do your symptoms last? | | | | | | | | | | | |  | | | | | |
|  | 1. More than four days a week | | | | | | | | | | | |  | | | | | |
|  | 1. More than four days a weeks in a row | | | | | | | | | | | |  | | | | | |
| 13. | How do the symptoms affect you? | | | | | | | | | | | |  | | | | | |
|  | 1. Symptoms disturb your sleep | | | | | | | | | | | |  | | | | | |
|  | 1. Symptoms restrict your daily activities (sports, leisure, etc.) | | | | | | | | | | | |  | | | | | |
|  | 1. Symptoms restrict your participation in school or work | | | | | | | | | | | |  | | | | | |
|  | 1. Symptoms are troublesome to you. | | | | | | | | | | | |  | | | | | |
| 14. | How much do your nose or eye symptoms bother you? | | | | | | | | | | | |  | | | | | |
|  | Not at all  0 | 1 | | 2 | 3 | | 4 | 5 | | 6 | 7 | | 8 | | 9 | | | Very Much  10 |
| 15. | How much did this nose problem interfere with your daily activities? | | | | | | | | | | | | | | | | | |
|  | Not at all  0 | 1 | | 2 | 3 | | 4 | 5 | | 6 | 7 | | | 8 | 9 | | | Very Much  10 |
| 15. | In which of the past 12 months did this problem occur? | | | | | | | | | | | | | | | | | |
|  | - January | | - February | | | - March | | | - April | | | - May | | | | - June | | |
|  | - July | | - August | | | - September | | | - October | | | - November | | | | - December | | |

| *Thinking about a typical night in the last month . . .* | | | | | | | | | | |
| --- | --- | --- | --- | --- | --- | --- | --- | --- | --- | --- |
| 1 | **How long does it take you to fall asleep?** | - 0–15 min | - 16–30 min | | - 31–45 min | | | - 46–60 min. | | - >60 min |
| 2 | **If you then wake up one or more times during the night, how long are you awake in total?** | - 0–15 min | - 16–30 min | | - 31–45 min | | | - 46–60 min. | | - >60 min |
| 3 | **If your final wake-up time occurs before you intend to wake up, how much earlier is this?** | I don’t wake up too early/Up to 15 min. early | - 16–30 min | | - 31–45 min | | | - 46–60 min. | | - >60 min |
| 4 | **How many nights a week do you have a problem with your sleep?** | - 0–1 | - 2 | | - 3 | | | - 4 | | - 5-7 |
| 5. | **How would you rate your sleep quality?** | - Very good | - Good | | - Average | | | - Poor | | - Very poor |
| *Thinking about the past month, to what extent has poor sleep . . .* | | | | | | | | | | |
| 6. | **What extent has poor sleep affected your mood, energy, or relationships?** | - Not   at all | | - A little | | - Somewhat | - Much | | - Very much | |
| 7. | **What extent has poor sleep affected your concentration, productivity, or ability to stay awake?** | - Not   at all | | - A little | | - Somewhat | - Much | | - Very much | |
| 8. | **What extent has poor sleep troubled you in general?** | - Not   at all | | - A little | | - Somewhat | - Much | | - Very much | |
| 9. | **How long have you had a problem with your sleep?** | - I don’t have a problem/<1month | | - 1–2 months | | - 3–6 months | - 7–12 months | | - >1 year | |
