# Supplementary material for: Allergic rhinitis: Disease characteristics and coping measures in Saudi Arabia
Source: PLoS One. 2019 Jun 26;14(6):e0217182. doi: 10.1371/journal.pone.0217182 (PMC6594581; doi:10.1371/journal.pone.0217182)
Supplement: S2 Survey — (DOCX) [file pone.0217182.s002.docx]

**دراسة مدى انتشار وحِدِّة و مسببات حساسية الأنف وآليات التغلب عليها في المملكة العربية السعودية**

١- العمر:

٢- الجنس :     
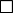
ذكر            
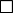
 أنثى

٣- الوزن:

٤- الطول:

٥- الجنسية :    
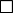
 سعودية      
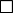
غير سعودية  (الرجاء التحديد …………………)

٦- مكان الإقامة: …………………..

٧- المهنة: ………………….

٧- الحالة الاجتماعية :     
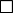
متزوج/ـه      
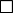
أعزب/عزباء

                             أرمل/ـه        
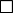
منفصل/ـه

٨- المستوى التعليمي:      غير متعلم     ثانوي

                             
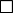
 جامعي        
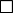
 دراسات عليا

**٩- الدخل الشهري :**
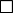
 مكتفي مادياً   
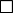
غير مكتفي مادياً

١٠- هل تعاني/ين من أي مشاكل صحية:

…………………………………………..

١١. -هل تعاني من الشخير :

نعم لا لا أعلم

١٢- مدخن/ـه :
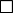
 نعم    ، عدد السيجائر في اليوم (ــــ)

                 
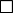
 لا

| **كل الأسئلة التالية حول مشاكل تحدث حينما لا تكون مصاباً بالزكام أو البرد**  ***خلال السنة الماضية:*** | | | | | | | | | | | | | | | | |
| --- | --- | --- | --- | --- | --- | --- | --- | --- | --- | --- | --- | --- | --- | --- | --- | --- |
| **1** | **هل تعاني من أحد الأعراض التالية، أو سبق أن عانيت منها** | | | | | | | | | | | |  | | |  |
| **أ.** | **سيلان الأنف** | | | | | | | | | | | | **☐ نعم** | | | **☐ لا** |
| **ب.** | **العطاس** | | | | | | | | | | | | **☐ نعم** | | | **☐ لا** |
| **ج.** | **انسداد الأنف (الإحساس بعدم القدرة على التنفس من الأنف)** | | | | | | | | | | | | **☐ نعم** | | | **☐ لا** |
| **د.** | **حكة الأنف** | | | | | | | | | | | | **☐ نعم** | | | **☐ لا** |
| **هـ.** | **إحمرار وحكة العينين، وزيادة الدموع** | | | | | | | | | | | | **☐ نعم** | | | **☐ لا** |
| **2** | **ماهو سبب هذه الأعراض** | | | | | | | | | | | | | | | |
| **أ.** | **لقاح الأشجار أو الزهور أو الأعشاب** | | | | | | | | | | | |  | | | |
| **ب.** | **الفطريات والعفن (داخلي أو خارجي)** | | | | | | | | | | | |  | | | |
| **ج.** | **الحيوانات صاحبة الفرو (الكلاب، القطط ...الخ)** | | | | | | | | | | | |  | | | |
| **د.** | **الغبار** | | | | | | | | | | | |  | | | |
| **3** | **كم استمرت هذه الأعراض** | | | | | | | | | | | | | | | |
| **أ.** | **اكثر من أربع أيام بالأسبوع** | | | | | | | | | | | |  | | | |
| **ب.** | **أكثر من أربع أيام بالأسبوع (على التوالي؟)** | | | | | | | | | | | |  | | | |
| **4** | **كيف أثرت هذه الأعراض على حياتك؟** | | | | | | | | | | | | | | | |
| **أ.** | **سببت اضطرابات في نومي** | | | | | | | | | | | |  | | | |
| **ب.** | **قيدت نشاطاتي اليومية** | | | | | | | | | | | |  | | | |
| **ج.** | **قيدت حضوري وإنتاجيتي في العمل/المدرسة** | | | | | | | | | | | |  | | | |
| **د.** | **الأعراض أتعبتني وازعجتني بشكل كبير** | | | | | | | | | | | |  | | | |
| **5** | **لأي مدى ضايقتك أعراض الأنف والعينين؟** | | | | | | | | | | | | | | | |
| 1 | | 2 | | **3** | **4** | | **5** | **6** | **7** | | **8** | **9** | | | **10** | |
| **6** | **لأي مدى أثرت مشاكل الأنف على نشاطاتك اليومية** | | | | | | | | | | | | | | | |
| 1 | | 2 | | **3** | **4** | | **5** | **6** | **7** | | **8** | **9** | | | **10** | |
| **7** | **في أي أشهر من السنة أصابتك هذه الأعراض** | | | | | | | | | | | | | | | |
| **محرم** | | | **صفر** | | | **ربيع الأول** | | **ربيع الثاني** | | **جمادى الأولى** | | | | **جمادى الثاني** | | |
| **رجب** | | | **شعبان** | | | **رمضان** | | **شوال** | | **ذو القعدة** | | | | **ذو الحجة** | | |

**١٤-هل أحد من أفراد العائلة يعاني من نفس الأعراض: نعم لا لا أعلم**

**١٥.١ ما هي الوسائل التي تستخدمها او الطرق التي تلجأ إليها لتخفيف هذه الأعراض ( اعشاب معينة، أدوية معينة، مكملات معينة، طرق منزلية، … الخ) اذكرها جميعها:**

**....................................................................................................................................................**

**١٥.٢ الرجاء تحديد مدى فعالية هذه الوسائل في تخفيف الاعراض ، علما بأن ١ اقل فعالية ، ١٠ اكثر فعالية.**

| **1** | **2** | **3** | **4** | **5** | **6** | **7** | **8** | **9** | **10** |
| --- | --- | --- | --- | --- | --- | --- | --- | --- | --- |

| **: في الليالي الماضية** | | | | | | |
| --- | --- | --- | --- | --- | --- | --- |
| **>٦٠**  **دقيقة** | **٤٦-٦٠**  **دقيقة** | **٣١-٤٥**  **دقيقة** | **١٦-٣٠**  **دقيقة** | **٠-١٥**  **دقيقة** | **كم من الوقت يستغرق منك الاستغراق في النوم؟** | ١ |
| **>٦٠**  **دقيقة** | **٤٦-٦٠**  **دقيقة** | **٣١-٤٥**  **دقيقة** | **١٦-٣٠**  **دقيقة** | **٠-١٥**  **دقيقة** | **إذا إستيقظت مرة أو أكثر خلال الليل ، كم مجموع الدقائق  التي تكون فيها مستيقظاً ؟** | ٢ |
| **>٦٠**  **دقيقة** | **٤٦-٦٠**  **دقيقة** | **٣١-٤٥**  **دقيقة** | **١٦-٣٠**  **دقيقة** | لا أستيقظ مبكراً\ إلى ١٥ دقيقة | **كم دقيقة تستيقظ مبكراً قبل موعدك المعتاد؟** | ٣ |
| ٥-٧ | ٤ | ٣ | ٢ | **٠-١** | **كم مرة في الاسبوع تعاني من مشاكل في النوم ؟** | ٤ |
| ضعيف جداً | ضعيف | معتدل | جيد | جيد جداً | **كيف تقيم جودة نومك ؟** | ٥ |
| **خلال الشهر الماضي، أي مدى كانت قلة النوم ...** | | | | | | |
| **كثير جداً** | **كثيراً** | **ى حدٍ ما** | **قليلاً** | **لا على الإطلاق** | **هل قلة النوم تؤثر على مزاجك ، نشاطك و علاقاتك ؟** | ٦ |
| **كثير جداً** | **كثيراً** | **ى حدٍ ما** | **قليلاً** | **لا على الإطلاق** | **هل قلة النوم تؤثر على تركيزك ، إنتاجيتك و القدرة على البقاء مستيقظاً؟** | ٧ |
| **كثير جداً** | **كثيراً** | **ى حدٍ ما** | **قليلاً** | **لا على الإطلاق** | **هل تضايقك قلة النوم بشكلٍ عام ؟** | ٨ |
| **<سنة** | **٧-١٢**  **أشهر** | **٣-٦**  **أشهر** | **١-٢**  **أشهر** | **لا أعاني من أى مشكلة/**  **< اقل من شهر** | **منذ متى وأنت تعاني من مشاكل في نومك ؟** | ٩ |
